# Association Between Smartphone Application Diversity and Occupational Participation Among Community-Dwelling Older Adults: A Cross-Sectional Study

**DOI:** 10.1007/s44474-026-00007-1

**Published:** 2026-09-14

**Authors:** Suguru Shimokihara, Kazuki Yokoyama, Hikaru Ihira, Yuriko Matsuzaki-Kihara, Atsushi Mizumoto, Hideyuki Tashiro, Hidekazu Saito, Keitaro Makino, Shunpei Katsuura, Masayuki Kobayashi, Kiyotaka Shimada, Kosuke Yama, Ryo Miyajima, Takeshi Sasaki, Nozomu Ikeda

**Affiliations:** 1 Graduate School of Biomedical Sciences, Health Sciences, Nagasaki University, Nagasaki, Japan; 2 Department of Occupational Therapy, School of Health Sciences, Sapporo Medical University, Sapporo, Japan; 3 Department of Physical Therapy, School of Health Sciences, Sapporo Medical University, Sapporo, Japan; 4 Department of Rehabilitation, Japan Healthcare University, Sapporo, Japan; 5 Major of Physical Therapy, Department of Rehabilitation, Faculty of Healthcare and Science, Hokkaido Bunkyo University, Eniwa, Japan; 6 Department of Health and Physical Education, Graduate School of Education, Hokkaido University, Sapporo, Japan; 7 Sapporo Medical University Hospital, Sapporo, Japan; 8 Graduate School of Health Sciences, Sapporo Medical University, Sapporo, Japan; 9 Ebetsu city hospital, Ebetsu, Japan

**Keywords:** Digital engagement, Digital inclusion, Gerontechnology, Occupational participation, Productivity

## Abstract

**Purpose:**

As digital technologies become increasingly embedded in daily life, evidence remains limited regarding how smartphone use relates to occupational participation among community-dwelling older adults. This study examined the association between smartphone application diversity and occupational participation in later life.

**Methods:**

This cross-sectional study used health checkup data collected during 2024–2025. Occupational participation was assessed using the Self-Completed Occupational Performance Index (SOPI), which covers leisure, productivity, and self-care domains. Participants were classified into high application diversity (HAD) and low application diversity (LAD) groups based on the number of 12 smartphone applications used daily. Group differences were tested, and adjusted regression models with bootstrap resampling were used to assess independent associations and robustness.

**Results:**

Among 97 participants, 67 were classified into the HAD group and 30 into the LAD group. Participants with HAD had higher total SOPI scores than those with LAD. In domain-specific analyses, application diversity was independently associated only with productivity-related occupational participation (adjusted β = 2.17, 95% CI: 0.49–3.86), and this association was supported by bootstrap results.

**Conclusions:**

Greater diversity in smartphone application use was associated with more favorable engagement in productivity-related occupations among community-dwelling older adults. These findings suggest that application diversity may be a meaningful indicator of how digital technologies are integrated into productive roles and responsibilities in later life.

**Supplementary Information:**

The online version contains supplementary material available at https://doi.org/10.1007/s44474-026-00007-1.

## Introduction

Population aging is accelerating worldwide, making the promotion of health, participation, and well-being among community-dwelling older adults a key public health and rehabilitation priority [1]. Along with demographic shifts, rapid digitalization has transformed daily life by positioning smartphones as essential tools for communication, information access, health management, and social participation. Smartphone adoption among older adults has increased substantially in developed countries. In Japan, 2023 national surveys reported that over 70% of adults aged 65 years own smartphone [2]. Similarly, survey data from Korea show that more than three-quarters of adults aged 60 years and older are smartphone owners [3]. In addition, reports from Europe indicate that smartphone use among older adults has become common in multiple high-income countries, with approximately 63–75% of adults aged 55–74 years [4]. These findings are consistent with a systematic review reporting that smartphone ownership among older adults in the United States exceeded 60% in 2021 [5]. This convergence of population aging and mobile technology expansion underscores the growing importance of understanding how smartphone use relates to health and occupational participation in later life.

Despite this rapid diffusion, ownership does not necessarily equate to effective or purposeful use. Previous studies have often operationalized smartphone use using binary indicators (e.g., users vs. non-users) or crude measures, such as frequency or duration of use [6]. However, these approaches may obscure heterogeneity in how older adults engage with smartphones in their daily lives. Evidence suggests that many older adults primarily use a limited set of basic applications (e.g., phone calls or text messaging), whereas more advanced or functionally supportive applications remain underutilized [7]. One approach to characterizing smartphone use is to consider the diversity of applications used in daily life rather than ownership or total screen time alone. Recent research has similarly conceptualized smartphone app usage diversity as the breadth of engagement across different application functions and demonstrated that its association with quality of life may vary according to age and digital literacy [8]. In the present study, application diversity refers simply to the number of different smartphone applications used in daily life and is used as an operational indicator of the breadth of digital engagement. It is not intended as a validated psychometric measure or a newly developed scale. Rather, this behavioral measure allows us to examine whether broader use of smartphone functions is associated with participation in everyday occupations. Previous research has shown that patterns of application use are associated with individual characteristics, including cognitive ability, supporting the potential relevance of application-use patterns when examining digital engagement in older adults [9].

From an occupational therapy perspective, digital technologies, such as smartphones, can be conceptualized as tools that enable and mediate occupational participation by supporting the performance of everyday activities. A scoping review of the digitalization of activities of daily living highlighted that everyday technologies, including everyday information and communication technologies, can function as both facilitators and barriers to older adults’ occupations [10]. This dual role of technology aligns with occupational therapy frameworks that conceptualize participation as an outcome shaped by person–environment–occupation interactions [11]. In older adults, occupational participation, defined as engagement in meaningful activities across leisure, productivity, and self-care domains, is particularly important for maintaining physical, cognitive, and psychological health, as well as for sustaining independence and quality of life [12, 13]. The Canadian model of occupational performance and engagement (CMOP-E) [14] emphasizes that health is shaped not only by the types of occupations performed but also by perceived occupational control, balance, and satisfaction in daily activities. Within this framework, the diversity of smartphone application use may reflect an individual’s ability to flexibly select and integrate digital tools across multiple occupational contexts, thereby supporting the key CMOP-E constructs. However, empirical evidence directly linking everyday digital behaviors, specifically patterns of smartphone application use, to occupational participation among older adults remains limited.

Therefore, this study aimed to examine the association between daily smartphone application diversity and occupational participation among community-dwelling older adults. We hypothesized that individuals who use a greater variety of smartphone applications in their daily lives would demonstrate higher levels of occupational participation across the leisure, productivity, and self-care domains. By focusing on application diversity as a behavioral indicator of everyday digital engagement, this study seeks to inform contemporary approaches to support occupational participation among older adults by clarifying how real-world smartphone use is linked to meaningful occupational participation in later life, with potential implications for digital inclusion strategies and community-based interventions for older adults in a digitalized society.

## Material and Methods

### Research Design

This cross-sectional study used data from the 2024 and 2025 assessment waves of the Widely Hokkaido Individual Training for Elderly (WHITE) Study, a community-based health check-up program targeting community-dwelling older adults in *ANONYMIZED FOR REVIEW*. The WHITE Study comprises questionnaire-based assessments and objective measurements of physical and cognitive functions conducted annually.

### Participants

**Fig. 1:**
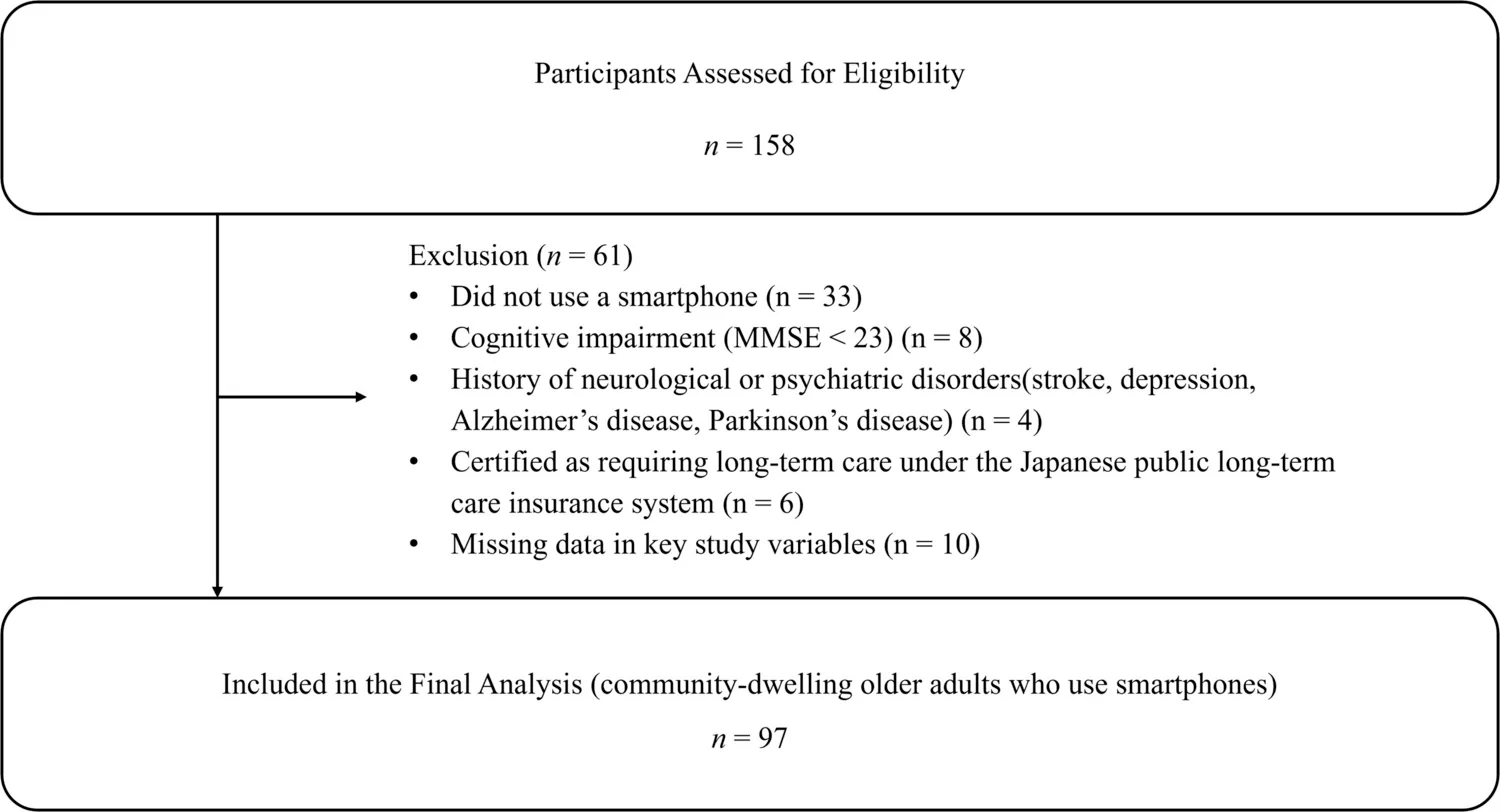
Study Flowchart. MMSE, mini-mental state examination

In total, 158 individuals participated in the 2024 and 2025 surveys. Participants were excluded if they (i) did not use a smartphone (n = 33), (ii) had cognitive impairment defined as a mini-mental state examination (MMSE) score < 23 (n = 8), (iii) had a history of neurological or psychiatric disorders, including stroke, depression, Alzheimer’s disease, or Parkinson’s disease (n = 4), (iv) had been certified as requiring long-term care under the Japanese public long-term care insurance system (n = 6), or (v) had missing data in key study variables (n = 10). After applying these exclusion criteria, 97 community-dwelling older adults who used smartphones in their daily lives were included in the final analysis. A flowchart summarizing the study is depicted in Fig. 1. All participants provided written informed consent and the WHITE Study protocol was approved by *ANONYMIZED FOR REVIEW*.

### Assessment of Smartphone Application Diversity

Participants were asked to indicate whether they used each smartphone application on a daily basis (yes/no) using a predefined list of commonly used applications. The list included phone calls, email, camera, quick response (QR) code use, web browsing, video streaming, healthcare-related applications, pedometer, text messaging, Facebook, X (formerly Twitter), and others. The “Other” category was coded as “used” if participants reported use of any smartphone application not included in the predefined list. For each participant, the number of applications reported was summed to generate a smartphone application diversity score, with higher scores indicating greater diversity of application use.

In this study, smartphone application diversity was operationalized as the number of different smartphone applications used, reflecting the breadth of digital engagement rather than the frequency or duration of use. Participants were categorized into two groups based on their application count: low application diversity (fewer than five applications; LAD) and high application diversity (five or more applications; HAD). The thresholds for the five applications were determined based on both empirical distribution within the present sample and prior population-level evidence.

Prior survey data from the United States showed that approximately 50% of older adults use three smartphone applications, with usage clustering of around five to six applications among approximately 40% of older smartphone users [15]. Based on this distribution, we adopted the use of five applications as a threshold to represent the greater diversity in smartphone application use among older adults. Therefore, a cutoff of five applications was considered a reasonable indicator of diverse and multifunctional smartphone use. In the current study, the median number of smartphone applications used was also five, which is consistent with the threshold derived from prior survey data. This concordance supports the appropriateness of using five applications as a cutoff to represent application-use diversity in the present study.

### Assessment of Occupational Participation

Occupational participation was assessed using the self-completed occupational performance index (SOPI) [16], a self-administered questionnaire based on the CMOP-E. The SOPI evaluates participation in meaningful occupations across the leisure, productivity, and self-care domains. Each domain assessed the perceived occupational control, occupational balance, and satisfaction with performance. Occupational control refers to the extent to which individuals feel able to decide when and how to engage in the occupation. Occupational balance reflects whether individuals perceive that they can allocate sufficient time and energy to the occupation within their daily lives. Satisfaction with performance captures the individual’s subjective appraisal of how satisfactorily they are able to perform the occupation. Each item is rated on a 5-point Likert scale. Each occupational domain comprises three items, yielding a domain score ranging from 3 to 15. The raw total score across the nine items ranges from 9 to 45 and can be standardized to a 0–100 scale using the formula (raw total score − 9)/36 × 100. In the present study, the standardized 0–100 score was used for the overall SOPI score, whereas the raw domain scores (range, 3–15) were used for the leisure, productivity, and self-care domains. Higher scores indicate more favorable occupational participation. In the original validation study, the SOPI showed good internal consistency, with a Cronbach’s α of 0.93, and moderate correlations among the three occupational categories (r = 0.50–0.64) [16]. In the present study, total SOPI scores and domain-specific scores for leisure, productivity, and self-care were used as outcome variables.

### Covariates

The demographic characteristics included age, sex, educational attainment, living arrangements, medication use, and employment status. Educational attainment was assessed by highest completed level (junior high school, high school, vocational school/junior college, or university/graduate school) and dichotomized as ≤ 12 or ≥ 13 years of education. Living arrangements were coded as living alone or with others, and medication use was recorded as a binary variable (yes/no). Employment status was categorized as remunerative work, volunteer work, or unemployed.

The smartphone-related variables included years of smartphone use and frequency of daily smartphone use. Participants were first asked whether they currently owned a smartphone, and only those who reported smartphone ownership were asked to indicate the number of years they had used a smartphone. No specific technical definition of a smartphone was provided in the questionnaire, and smartphone ownership was based on participants’ self-report. Reported duration of smartphone use ranged from 1 to 18 years, and no values were considered implausible or excluded on the basis of duration. Daily smartphone use frequency was assessed using a nine-category scale ranging from “almost never” to “ ≥ 5 h per day” and subsequently dichotomized as < 1 h or ≥ 1 h per day, consistent with prior studies examining smartphone or digital device use intensity among older adults [17, 18].

Physical and cognitive functions were assessed using established and widely used measures. Gait speed was assessed using the 10-m walk test. Participants were instructed to walk at their usual comfortable pace, and gait speed was calculated as the distance walked (10 m) divided by the time required to complete the test and expressed in meters per second (m/s) [19]. Gait speed is a validated indicator of physical function and mobility in older adults and has been associated with functional independence and participation in daily activities [20]. Cognitive function was assessed using the MMSE [21], a standard screening tool for global cognitive status. Functional decline was evaluated using the Kihon checklist (KCL) [22], a comprehensive screening instrument commonly used in Japan to assess frailty-related domains, including physical, cognitive, and social functioning. Depressive symptoms were assessed using the 15-item geriatric depression scale (GDS-15) [23], a validated self-report measure frequently employed in community-dwelling older populations.

These variables were selected as potential confounders because they may be independently associated with occupational participation and could influence patterns of smartphone use, thereby potentially confounding the observed association between application diversity and occupational participation.

### Statistical Analysis

Descriptive statistics were calculated for all the variables. Differences in SOPI scores between the LAD and HAD groups were examined using Welch’s t-test and Pearson’s Chi-square test.

Based on the results of the preliminary two-group comparisons, generalized linear models (GLMs) were constructed to examine whether application diversity was independently associated with occupational participation. SOPI subdomain scores that demonstrated statistically significant differences between application diversity groups were entered as dependent variables, with the application diversity group as the independent variable, adjusted for age, sex, education, living situation, medication, employment status, smartphone usage years, daily smartphone use time, gait speed, GDS-15, KCL, and MMSE scores.

Given the limited sample size, bootstrap resampling (2,000 iterations) was additionally conducted to assess the robustness of the regression coefficients. Statistical significance was set at p < 0.05. All analyses were performed using R software. Given the exploratory nature of these analyses, p-values were not adjusted for multiple comparisons.[24] All statistical analyses were conducted using R version 4.2.2 (R Foundation for Statistical Computing, Vienna, Austria), with the significance level set at p < 0.05.

## Results

### Characteristics of Study Participants

The participant characteristics by application diversity group are shown in Table 1. Ninety-seven community-dwelling older adults were included in the analysis (median age, 77.0 years; interquartile range [IQR], 75.0–82.0), of whom 73% were female. Regarding employment status, 12 participants (12%) were engaged in remunerative work, 13 (13%) in volunteer work, and 72 (74%) were unemployed. Employment status differed significantly between the application diversity groups (p = 0.048), with remunerative and volunteer work being more common in the HAD group. The median usage of smartphone was 7.0 years (IQR, 4.0–10.0), and 66% of participants reported using smartphones for < 1 h per day. Based on smartphone application diversity, participants were classified into LAD group, defined as those using fewer than five applications (n = 30), and HAD group, defined as those using five or more applications (n = 67).

**Table 1:** Baseline Characteristics of Participants Based on Application Diversity

Characteristic	Overall n = 97^1^	Lower application diversity n = 30^1^	Higher application diversity n = 67^1^	p-value^2^
Age	77.0 (75.0–82.0)	80.5 (77.0–85.0)	76.0 (74.0–80.0)	< 0.001
Sex				0.60
Female	71(73%)	23(77%)	48(72%)	
Male	26(27%)	7(23%)	19(28%)	
Education years				0.084
≤ 12 years	52(54%)	20(67%)	32(48%)	
> = 13 years	45(46%)	10(33%)	35(52%)	
Living alone	41(42%)	10(33%)	31(46%)	0.20
Medication	77(80%)	22(76%)	55(82%)	0.50
Employment status				0.048
Remunerative work	12(12%)	2(7%)	10(15%)	
Volunteer work	13(13%)	1(3%)	12(18%)	
Unemployed	72(74%)	27(90%)	45(67%)	
Smartphone usage years	7.0 (4.0–10.0)	6.0 (4.0–10.0)	8.0 (5.0–12.0)	0.20
Daily smartphone use frequancy				0.016
< 1 h	64(66%)	25(83%)	39(58%)	
> = 1 h	33(34%)	5(17%)	28(42%)	
Gait speed	1.4 (1.3–1.5)	1.4 (1.2–1.5)	1.4 (1.3–1.6)	0.30
MMSE	29.0 (26.0–30.0)	28.0 (26.0–29.0)	29.0 (26.0–30.0)	0.061
GDS-15	3.0 (2.0–5.0)	4.0 (3.0–6.0)	3.0 (1.0–4.0)	0.043
KCL	5.0 (3.0–6.0)	6.0 (4.0–8.0)	4.0 (2.0–6.0)	0.016

^1^Median (Q1-Q3); n (%)

^2^Welch Two Sample t-test; Pearson's Chi-squared test

Abbreviations: GDS-15; 15-item Geriatric Depression Scale, KCL; Kihon check-list, MMSE; mini-mental state examination

Compared with the LAD group, participants in the HAD group were significantly younger (p < 0.001) and more likely to report daily smartphone use of ≥ 1 h (p = 0.016). In contrast, the LAD group demonstrated higher GDS-15 scores and higher Kihon checklist KCL scores than that of the HAD group (p = 0.043 and p = 0.016, respectively). No significant between-group differences were observed in terms of sex, educational attainment, living arrangements, medication use, medical history, MMSE, or gait speed.

### Smartphone Application Use Patterns

**Fig. 2:**
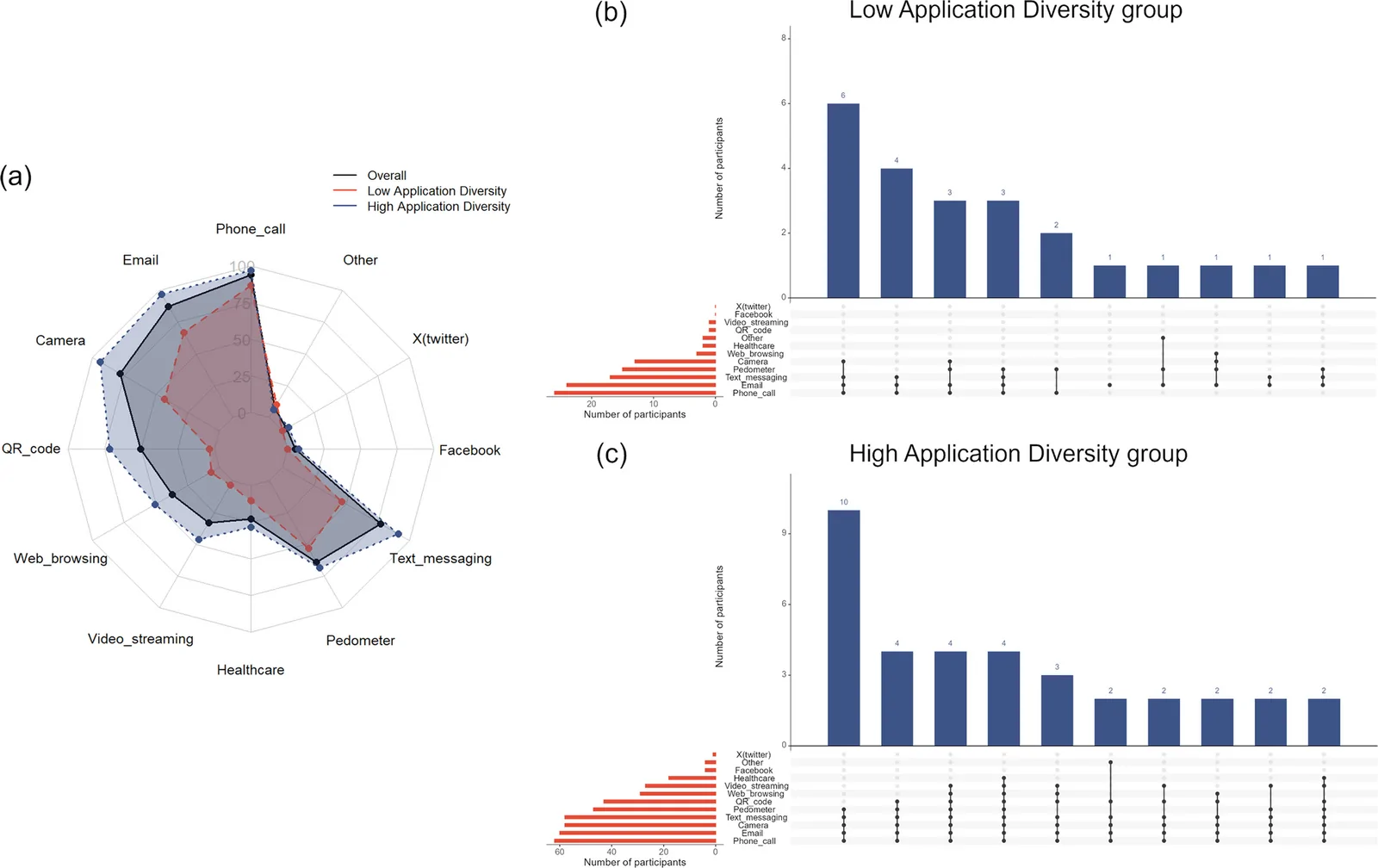
Distribution and Combinations of Smartphone Application Use Among Participants (**a**) The radar chart presents the percentage of participants reporting daily use of each smartphone application in the overall sample, lower application diversity group (< 5 applications), and higher application diversity group (≥ 5 applications). (**b**) The plot presents the 10 most frequent application-use combinations in the lower application diversity group. (**c**) The plot presents the corresponding 10 most frequent combinations in the higher application diversity group. In panels (**b**) and (**c**), the vertical bars represent the number of participants with each application-use pattern, the connected dots below each bar indicate the applications comprising that pattern, and the horizontal bars represent the total number of participants using each application

Figure 2 presents the distribution and combinations of smartphone applications used daily by the participants. Figure 2a shows that basic communication-related applications, including phone calls (94%), email (88%), text messaging (78%), and camera (77%), were the most commonly used across the entire sample. Compared with the LAD group, participants in the HAD group showed higher usage rates across a broader range of application categories, including web browsing, camera, QR codes, and healthcare-related applications. In contrast, application use in the LAD group was largely concentrated on communication-focused functions, with lower engagement observed in non-communication applications. Figure 2b illustrates the 10 common combinations of smartphone applications used in the LAD group. In this group, the most frequent combinations were largely limited to basic communication-related applications, particularly phone calls, email, and text messaging, which were either used alone or in simple combinations with one additional application. Figure 2c presents the corresponding application combinations for the HAD group. In contrast to the LAD group, the HAD group exhibited a wider variety of application combinations in which basic communication applications were commonly used alongside multiple additional applications such as web browsing, camera, QR code use, healthcare-related applications, and video streaming.

### Association Between Application Diversity and Occupational Participation

**Fig. 3:**
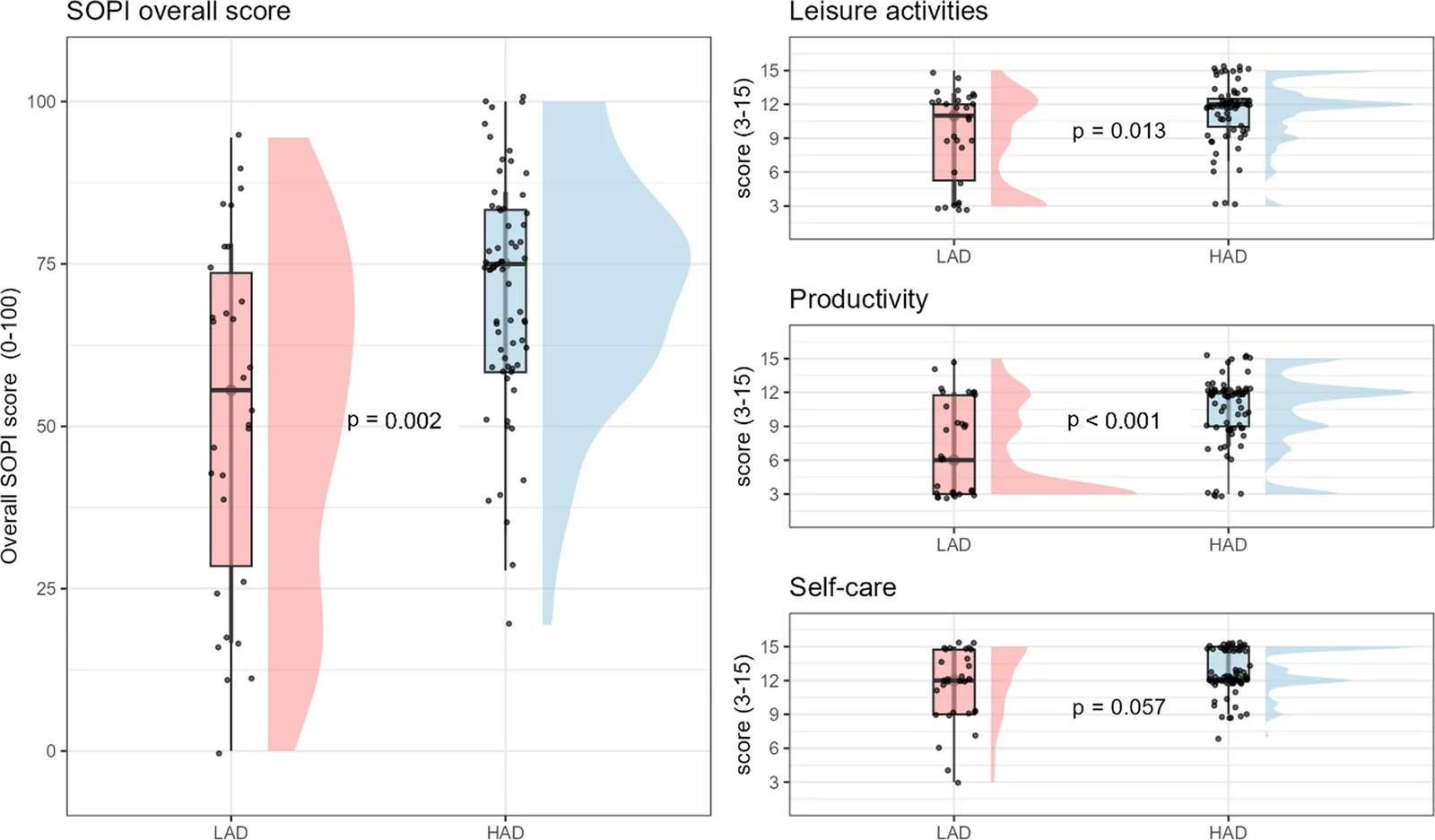
Overall and domain-specific SOPI scores according to smartphone application diversity

Figure 3 compares the SOPI scores between the two application diversity groups. The HAD group demonstrated significantly higher overall SOPI scores than that of the LAD group (median [IQR]: 75.0 [58.3–83.3] vs. 55.6 [25.0–75.0], p = 0.002). When the SOPI scores were examined by occupational domain, significant between-group differences were observed in both the leisure and productivity domains. The HAD group had higher leisure scores than that of the LAD group (12.0 [10.0–13.0] vs. 11.0 [5.0–12.0], p = 0.013), and a more pronounced difference was observed for productivity scores (12.0 [9.0–12.0] vs. 6.0 [3.0–12.0], p < 0.001). In contrast, no statistically significant difference was found between the groups in the self-care domain (p = 0.057).

Participants were classified into the lower application diversity group (LAD; < 5 applications used daily) and higher application diversity group (HAD; ≥ 5 applications used daily). The overall SOPI score ranges from 0 to 100, whereas the leisure activities, productivity, and self-care domain scores each range from 3 to 15. Higher scores indicate more favorable occupational participation. Individual observations, boxplots, and score distributions are shown. Between-group differences were examined using Welch’s t-test. Abbreviation: SOPI; self-completed occupational performance index.

To examine whether application diversity was independently associated with occupational participation, GLMs were constructed with the SOPI leisure and productivity subscale scores as dependent variables. The application diversity group was entered as the primary independent variable, adjusted for age, sex, education, living alone, medication use, employment status, years of smartphone use, daily smartphone use time, gait speed, and MMSE, KCL, and GDS-15 scores. In the adjusted models, application diversity was not significantly associated with SOPI leisure scores (regression coefficient = 0.66, 95% CI: − 0.89 to 2.22). Full results of the leisure model are provided in Supplementary Table S1. By contrast, using five or more smartphone applications was independently associated with higher SOPI productivity scores (regression coefficient = 2.17, 95% confidence interval [CI]: 0.49–3.86). The GLM results for the SOPI productivity subdomain are presented in Table 2.

**Table 2:** Estimation of Values from the Original and 2000 Bootstrap Samples of the GLM

Independent variables	Original GLM			GLM summary of 2000 bootstrap samples			
	Estimate	SE	95% CI (lower, upper)	Median estimate	SE	Bias	95% CI (lower, upper)
(Intercept)	14.02	9.25	−4.12, 32.15	13.95	9.84	0.35	−3.60, 35.19
Application diversity (ref: lower application diversity)	2.17*	0.86	0.49, 3.86	2.27*	0.99	0.08	0.16, 4.07
Age	−0.05	0.07	−0.19, 0.10	−0.04	0.08	0	−0.24, 0.09
Sex (ref: female)	−1.83	0.97	−3.73, 0.06	−1.93	1.02	−0.10	−3.66, 0.22
Education years (ref: ≤ 12 years)	0.52	0.53	−0.52, 1.56	0.54	0.54	0.01	−0.65, 1.58
Living alone	−1.26	0.82	−2.86, 0.34	−1.28	0.83	0	−2.88, 0.42
Medication	−0.69	0.63	−1.92, 0.53	−0.68	0.61	0.02	−1.99, 0.54
Remunerative work (ref: Unemployed)	0.16	1.14	−2.07, 2.39	0.11	1.1	0.01	−1.80, 2.50
Volunteer work (ref: Unemployed)	2.13*	1.08	0.02, 4.24	2.11*	0.86	−0.03	0.41, 3.84
Smartphone usage years	0.04	0.06	−0.09, 0.16	0.04	0.06	0	−0.09, 0.16
Daily smartphone use frequancy (ref: < 1 h)	0.19	0.75	−1.28, 1.66	0.18	0.80	0	−1.35, 1.76
Gait speed	2.46	1.93	−1.31, 6.24	2.51	2.11	0.03	−1.85, 6.51
MMSE	−0.06	0.20	−0.45, 0.33	−0.07	0.20	−0.02	−0.48, 0.31
KCL	−0.1	0.15	−0.38, 0.19	−0.09	0.16	0	−0.43, 0.21
GDS-15	−0.36*	0.16	−0.66, −0.05	−0.37*	0.16	−0.01	−0.64, −0.02

* p < 0.05

The dependent variable was the productivity subdomain score of the Self-Completed Occupational Performance Index.

### Robustness Analyses

Given the modest sample size, bootstrap resampling (2,000 iterations) was conducted to assess the robustness of the association between application diversity and productivity. The bootstrap analysis yielded a median regression coefficient of 2.24, with a 95% CI of 0.49–3.86, confirming the stability of the observed association. Table 2 presents the description and distribution of the regression coefficients obtained via bootstrapping.

## Discussion

This study examined the association between smartphone application diversity and occupational participation among community-dwelling older adults. The findings indicated that older adults who used a greater variety of smartphone applications demonstrated significantly higher overall occupational participation, with the association driven primarily by the productivity domain. This relationship remained robust after adjusting for age, functional abilities, sociodemographic factors, and smartphone usage/duration, and was further supported by bootstrap analyses. Taken together, the present results indicate that variation in how older adults utilize smartphone applications is associated with more active participation in productive activities and offer additional insight into approaches for supporting occupational participation among older adults.

The participants’ median age exceeded the late seventies, reflecting the demographic reality of aging communities in Japan. Despite the advanced age of the study population, smartphone ownership appeared to be widespread among the older age groups, reflecting the increasing penetration of mobile technologies in later life. However, the observed variability in application diversity highlights the persistent digital heterogeneity of smartphone use. Participants with lower application diversity tended to be older and used smartphones for shorter durations, consistent with prior reports indicating that aging is associated with narrower digital engagement and reduced functional use of information and communication technologies [25, 26]. These characteristics are important when interpreting the findings, as they suggest that application diversity may reflect not only individual preferences but also age-related changes in the cognitive, physical, and experiential factors that shape digital engagement. Longitudinal and population-based studies have linked cognitive function to the use of information and communication technology in later life, indicating that cognitive capacity is an important condition for initiating and sustaining digital engagement [27]. Other studies using objective indicators of impairment have shown that physical and cognitive limitations are associated with the non-use of digital services and lower digital competence, highlighting the role of functional constraints in shaping everyday technology use [28]. Beyond functional capacity, prior syntheses emphasize experiential and contextual factors, such as prior exposure to digital tools, skills, confidence, and the availability of support [29]. Collectively, these findings suggest that heterogeneity in smartphone application diversity may reflect not only individual preferences but also age-related functional changes and accumulated experiences that influence how digital tools are incorporated into daily life.

A key finding was that application diversity was associated with occupational participation in the productivity domain but not with leisure or self-care. This pattern may be partly explained by the increased use of applications such as email, camera, QR codes, web browsing, video streaming, healthcare, and text messaging in the HAD group. These applications may support productivity-related occupations by facilitating information exchange, task or event documentation, access to online services and resources, management of appointments or health-related information, and coordination of daily responsibilities with others. Productivity-related occupations typically involve planning, coordination, information management, and role fulfillment [30, 31], which can be directly supported by multifunctional smartphone use. Applications for communication, scheduling, navigation, and information retrieval may facilitate engagement in socially productive roles such as managing household responsibilities, volunteering, or supporting others. Empirical research supports this interpretation by showing that many older adults use digital methods for instrumental and productivity-related daily tasks, including managing finances, navigating the community, and organizing appointments or to-do activities [32]. Qualitative evidence further indicates that older adults can learn and adopt smartphone-based calendars and reminder systems to support the scheduling and completion of everyday activities, highlighting the practical relevance of mobile tools for planning and coordination [33]. In addition, a systematic review summarized that older adults use off-the-shelf smartphones and tablets, including native applications such as calendar functions and commercially available apps, to support cognition, memory, and activities of daily living, which are closely linked to effective role fulfillment and information management [5]. Notably, only 12% of participants were engaged in remunerative work, whereas 13% participated in volunteer work and 74% were unemployed. Although employment/volunteer status differed between the application diversity groups, the association between higher application diversity and productivity remained significant after adjustment for this variable. This suggests that the observed association is unlikely to reflect formal employment or volunteer participation alone, but may encompass a broader range of productive occupations, including household responsibilities and other roles involving planning, coordination, and responsibility.

In contrast, leisure and self-care activities may depend less on digital tools or may be maintained through established routines. This pattern is consistent with the CMOP-E, which conceptualizes technology as an environmental factor that enables occupational performance and participation. Accordingly, the extent to which technology is related to participation is expected to vary by occupational domain and by an individual’s perceived fit between available environmental support and the demands of specific occupations. Therefore, several alternative explanations must be considered. Reverse causation is possible, because individuals with greater productive role demands may adopt more applications to manage their daily tasks. Residual confounding related to socioeconomic resources, access, and informal support may also remain [29]. In addition, null findings for self-care may reflect a limited measurement fit, as these activities are less readily digitized than instrumental or organizational tasks [34]. Nonetheless, these findings suggest that application diversity may serve as a feasible marker of how everyday technologies are integrated into productivity-oriented occupations that support participation in later life [10].

The findings of this study have several implications for occupational therapy practices in community settings. First, assessing application diversity together with occupational participation may provide occupational therapists with a practical way to better understand the environmental factors that support or constrain older adults’ engagement in meaningful occupations in an increasingly digitalized society. Rather than focusing solely on ownership or overall screen time, clinicians may benefit from examining which applications are used in daily life, and how these patterns align with specific occupational demands and goals. Second, interventions that support purposeful and selective smartphone use, particularly for productivity-related activities, may facilitate participation in meaningful occupations among older adults; however, this possibility requires further empirical evaluation. In addition, higher smartphone proficiency was associated with higher-level competence [35], suggesting that supporting skill development may be relevant when designing occupation-centered digital interventions. Occupational therapists are well-positioned to develop individualized interventions that align smartphone use with clients’ values, routines, and roles. This approach may become increasingly important as digital technologies continue to mediate access to services, information, and social participation in aging societies.

## Llimitations

Some limitations of this study should be considered when interpreting the results. First, the sample size was relatively small, which may limit the statistical power and generalizability. Although bootstrap analyses were conducted to address this limitation, replication in a larger sample is warranted. In addition, 73% of the participants were female, which may limit the generalizability of the findings to older men. Future studies with more balanced sex distributions are needed to determine whether similar associations are observed across sexes. Second, its cross-sectional design precludes causal inferences. It remains unclear whether greater application diversity leads to higher occupational participation or whether individuals with higher occupational participation are more likely to adopt diverse smartphone applications. Longitudinal studies are required to clarify the directionality of this relationship. Third, smartphone-related variables, including application use and duration of smartphone use, were assessed using self-reported measures, which may have been subject to recall or misclassification bias. In particular, because a specific technical definition of a smartphone was not provided, some participants may have interpreted the duration-of-use question broadly and included previous use of feature phones. Although no clearly implausible values were observed in the reported duration of smartphone use, this possibility cannot be completely excluded. Objective measures of smartphone use, such as usage logs, can complement self-reported data in future research. Despite these limitations, this study provides novel insights into the relationship between digital engagement and occupational participation in later life.

## Conclusion

This study demonstrated that greater smartphone application diversity is associated with higher occupational participation among community-dwelling older adults, particularly in the productivity domain. These findings suggest that application diversity may serve as a meaningful indicator of the integration of digital technologies into everyday occupations. From an occupational therapy perspective, supporting older adults in expanding and purposefully using smartphone applications may improve their participation in productive roles and activities. Future research should employ longitudinal designs and larger samples to further elucidate the mechanisms linking digital engagement and occupational participation and to inform the development of occupation-centered digital interventions for aging populations.

## Supplementary Information

Below is the link to the electronic supplementary material.

ESM 1 (DOCX 25.9 KB)

## Data Availability

The data that support the findings of this study are available on request from the corresponding author, S.S. The data are not publicly available due to restrictions their containing information that could compromise the privacy of research participants.
