# Supplementary material for: Association Between Smartphone Application Diversity and Occupational Participation Among Community-Dwelling Older Adults: A Cross-Sectional Study
Source: Scand J Occup Ther. 2026 Sep 14;33(1):5. doi: 10.1007/s44474-026-00007-1 (PMC13574877; doi:10.1007/s44474-026-00007-1)
Supplement: Supplementary file 1 [file 44474_2026_7_MOESM1_ESM.docx]

**Supplementary materials**

**Supplementary Table S1. Adjusted Generalized Linear Model for the Self-Completed Occupational Performance Index Leisure Subdomain Score**

| Independent variables | Original GLM | | |
| --- | --- | --- | --- |
|  | Estimate | SE | 95% CI  (lower, upper) |
| (Intercept) | 17.07* | 8.55 | 0.31, 33.83 |
| Application diversity (ref: lower application diversity) | 0.66 | 0.79 | -0.89, 2.22 |
| Age | -0.09 | 0.07 | -0.23, 0.04 |
| Sex (ref: female) | 0.52 | 0.9 | -1.24, 2.27 |
| Education years (ref: ≤12 years) | 0.33 | 0.49 | -0.63, 1.29 |
| Living alone | 0.96 | 0.75 | -0.51, 2.44 |
| Medication | 1.15* | 0.58 | 0.02, 2.28 |
| Remunerative work (ref: Unemployed) | -1.65 | 1.05 | -3.71, 0.42 |
| Volunteer work (ref: Unemployed) | 0.2 | 0.99 | -1.75, 2.15 |
| Smartphone usage years | -0.01 | 0.06 | -0.13, 0.10 |
| Daily smartphone use frequancy (ref: <1hr) | 0.11 | 0.69 | -1.25, 1.47 |
| Gait speed | 0.7 | 1.78 | -2.79, 4.19 |
| MMSE | 0.01 | 0.19 | -0.35, 0.38 |
| KCL | -0.13 | 0.14 | -0.39, 0.14 |
| GDS-15 | -0.42** | 0.15 | -0.70, -0.13 |

* p < 0.05, ** p < 0.01, *** p < 0.001

The dependent variable was the leisure subdomain score of the Self-Completed Occupational Performance Index.

Abbreviations: CI; confidence interval, GDS-15; 15-item Geriatric Depression Scale, GLM; general liner model, KCL; Kihon check-list, MMSE; mini-mental state examination, SE; standard error.
